# Recombinant adeno-associated virus serotype 6 (rAAV2/6)-mediated gene transfer to nociceptive neurons through different routes of delivery

**DOI:** 10.1186/1744-8069-5-52

**Published:** 2009-09-08

**Authors:** Chris Towne, Marie Pertin, Ahmed T Beggah, Patrick Aebischer, Isabelle Decosterd

**Affiliations:** 1 Brain Mind Institute, Ecole Polytechnique Fédérale de Lausanne, EPFL, Lausanne, Switzerland; 2 Pain Research Unit, Department of Anesthesiology, Centre Hospitalier Universitaire Vaudois and University of Lausanne, Lausanne, Switzerland; 3 Department of Cell Biology and Morphology, University of Lausanne, Lausanne, Switzerland

## Abstract

**Background:**

Gene transfer to nociceptive neurons of the dorsal root ganglia (DRG) is a promising approach to dissect mechanisms of pain in rodents and is a potential therapeutic strategy for the treatment of persistent pain disorders such as neuropathic pain. A number of studies have demonstrated transduction of DRG neurons using herpes simplex virus, adenovirus and more recently, adeno-associated virus (AAV). Recombinant AAV are currently the gene transfer vehicles of choice for the nervous system and have several advantages over other vectors, including stable and safe gene expression. We have explored the capacity of recombinant AAV serotype 6 (rAAV2/6) to deliver genes to DRG neurons and characterized the transduction of nociceptors through five different routes of administration in mice.

**Results:**

Direct injection of rAAV2/6 expressing green fluorescent protein (eGFP) into the sciatic nerve resulted in transduction of up to 30% eGFP-positive cells of L4 DRG neurons in a dose dependant manner. More than 90% of transduced cells were small and medium sized neurons (< 700 μm^2^), predominantly colocalized with markers of nociceptive neurons, and had eGFP-positive central terminal fibers in the superficial lamina of the spinal cord dorsal horn. The efficiency and profile of transduction was independent of mouse genetic background. Intrathecal administration of rAAV2/6 gave the highest level of transduction (≈ 60%) and had a similar size profile and colocalization with nociceptive neurons. Intrathecal administration also transduced DRG neurons at cervical and thoracic levels and resulted in comparable levels of transduction in a mouse model for neuropathic pain. Subcutaneous and intramuscular delivery resulted in low levels of transduction in the L4 DRG. Likewise, delivery via tail vein injection resulted in relatively few eGFP-positive cells within the DRG, however, this transduction was observed at all vertebral levels and corresponded to large non-nociceptive cell types.

**Conclusion:**

We have found that rAAV2/6 is an efficient vector to deliver transgenes to nociceptive neurons in mice. Furthermore, the characterization of the transduction profile may facilitate gene transfer studies to dissect mechanisms behind neuropathic pain.

## Background

Chronic pain, and in particular, neuropathic pain, is a common condition that greatly impacts on individual quality of life and places considerable burden on the healthcare system as well as society [1,2]. Current pharmacological agents used for treating chronic pain act mainly through analgesic mechanisms not specific to the intrinsic pathways of pain and are prone to elicit side effects. An intricate understanding of the cellular mechanisms and pathways contributing to pain would therefore greatly facilitate the development of novel therapeutics towards a mechanisms-based pain management.

Gene transfer to primary sensory neurons within the nociceptive pathway is a novel approach to study pain. Transgenes of interest can be expressed in adult animals in order to dissect their roles within pain signaling pathways and are an alternative to transgenic approaches. Gene transfer to nociceptors is also a promising strategy for the management of chronic pain, allowing expression of a transgene at restricted sites in the nervous system therefore selectively targeting pain-related pathways without eliciting off-target effects.

Gene transfer to nociceptive neurons has been achieved through both viral and non-viral methods. Plasmid DNA driving expression of proteins have been delivered to sensory neurons via liposomes [3], electroporation [4] and delivery through hypertonic diluent [5] through peripheral or direct injections to the central nervous system. The major drawback of these methods is that they result in transient protein expression persisting no longer than two weeks. Alternatively, viruses can be used to drive longer transgene expression. The efficacy of viral-mediated gene delivery depends primarily on the type of delivery method and the type of virus being used. Adenovirus, herpes-simplex virus (HSV), lentivirus and adeno-associated virus (AAV) have been reported to deliver transgenes to nociceptive pathways through a number of delivery routes including subcutaneous [6], intramuscular [7], intraneural [8], intrathecal [9,10], intraspinal [11,12], direct dorsal root ganglia injections [13] and also topical applications of the virus in the case of HSV [14,15]. While these studies have resulted in transgene expression at favorable sites and with concomitant reduction in pain-related behavior, the transduction profile has not often been characterized. This is common in studies that utilize secreted transgenes that act in the extracellular environment, such as enkephalin, endomorphins and interleukins, where only a few transduced cells are required to deliver the transgene to the affected cellular neighborhood and modulate pain perception [16].

The aim of this study was to assess recombinant AAV (rAAV) serotype 6 as a gene transfer tool to target cellular mechanisms involved in the generation and development of chronic pain in mice. rAAVs are powerful gene transfer vectors due to their broad tissue tropism, efficient and stable transduction (> years), poor immunogenicity and ability to infect post-mitotic cells *in vivo *[17]. The serotype 6 vector (rAAV2/6) was chosen from the observation of sensory fiber transduction following intravenous delivery in previous experiments in mice [18] and the high tropism for neurons following direct injections into the central nervous system [19]. In the present study, we have delivered this vector through various routes of administration and precisely mapped and compared the transduction profiles obtained within the dorsal root ganglia (DRG) and spinal cord.

## Results

### rAAV2/6 transduces dorsal root ganglia and is dependant upon mode of delivery

**Figure 1:**
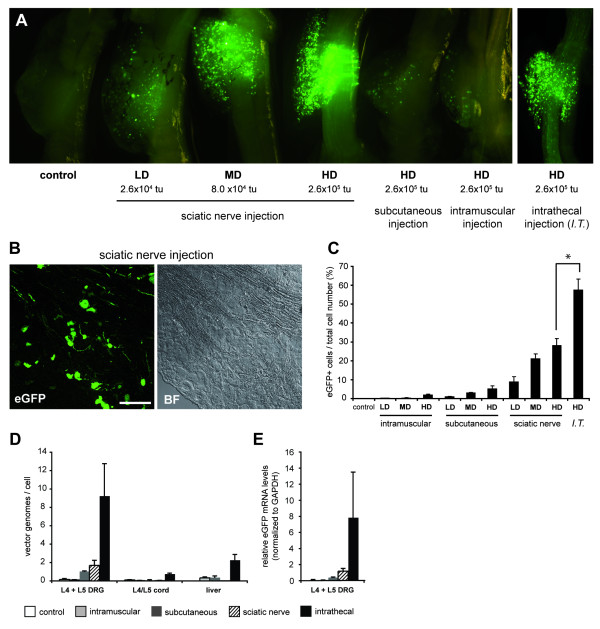
**rAAV2/6 transduction of the dorsal root ganglia following subcutaneous, intramuscular, sciatic nerve and intrathecal delivery**. (A) Macroscopic view of L4 DRG of mice injected with rAAV2/6 expressing eGFP. From left to right: uninjected, dose response of sciatic nerve injected, subcutaneous (high dose), intramuscular (high dose), intrathecal (high dose). (B) Native eGFP expression and corresponding bright field image in a typical 12 μm L4 DRG section following sciatic nerve delivery. Scale bar, 100 μm. (C) Transduction rates within L4 DRG following subcutaneous, intramuscular, sciatic nerve delivery at three doses (n = 3 per group and dose) and intrathecal delivery at the highest dose (n = 5) expressed as a percentage of eGFP expressing profiles per total number of DRG cells observed. LD, low dose (2.6 × 10^4 ^tu); MD, medium dose (8.0 × 10^4 ^tu); HD, high dose (2.6 × 10^5 ^tu). (D) qPCR against the vector genome (beta globin intron of rAAV2/6) in DRG, spinal cord and liver for the high dose of the four administration routes. (E) Reverse transcription qPCR against eGFP mRNA transcripts normalized with GAPDH mRNA in the DRG for the various protocols.

rAAV2/6 (rAAV serotype 2 genome packaged in the serotype 6 capsid) was delivered to C57BL/6 mice through four routes; subcutaneously at the middle and lateral plantar surface of the hind foot; intramuscularly in the triceps surae muscle of the hind limb; in the sciatic nerve; and intrathecally at the lumbar level. Three different doses of vector were examined (except for the intrathecal administration which received only the high dose) and the transduction was assessed three weeks later. All modes of delivery resulted in eGFP epifluorescence within the whole dissected L4 DRG demonstrating that rAAV2/6 is capable of transducing sensory neurons (Fig. 1A, 1B). The levels of transduction were dependant upon the mode of delivery (from highest to lowest) with intrathecal, sciatic nerve, subcutaneous and intramuscular delivery resulting in 57.5 ± 0.1, 28.1 ± 3.1, 3.5 ± 1.7 and 1.6 ± 0.6 percent of eGFP expressing profiles per total number of DRG cells observed, respectively. Furthermore, the transduction rate was dose dependent in instances where examined (Fig. 1C).

The relative transduction efficiencies for the various delivery routes were next assessed by quantifying the vector genome copy numbers per cell (vg/cell) following injection of the high dose of vector. Intrathecal administration resulted in the highest level of vector genomes in DRG (L4 + L5 combined) (9.2 ± 3.5 vg/cell), with sciatic nerve and subcutaneous delivery providing lower copy numbers (1.7 ± 0.6 vg/cell and 1.0 ± 0.5 vg/cell, respectively) (Fig. 1D). Vector genomes were not detected following intramuscular delivery. Intrathecal delivery also resulted in the presence of vector genomes in the lumbar spinal cord (0.8 ± 0.1 vg/cell) and gave the highest numbers of vector genomes in the liver (2.3 ± 0.6 vg/cell). Liver transduction is typical for rAAV2/6 and reflects leakage of the vector from the injection site into the blood. Interestingly, only the sciatic nerve injection failed to register vector genomes in the liver demonstrating the specificity of this injection technique.

Reverse transcription quantitative PCR (qPCR) against eGFP mRNA transcripts recapitulated this transduction profile. mRNA transcripts for eGFP were approximately seven times more abundant for intrathecal than sciatic nerve delivery in lumbar DRG (Fig. 1E). Subcutaneous delivery resulted in approximately one third of transcripts compared to sciatic nerve delivery and intramuscular administration failed to result in detectable levels of eGFP mRNA. Curiously, despite the presence of vector genomes in spinal cord and liver observed above, we did not detect any eGFP mRNA expression in these two tissues regardless of the delivery route (not shown).

### Efficient transduction of nociceptive neurons following sciatic nerve and intrathecal injections

**Figure 2:**
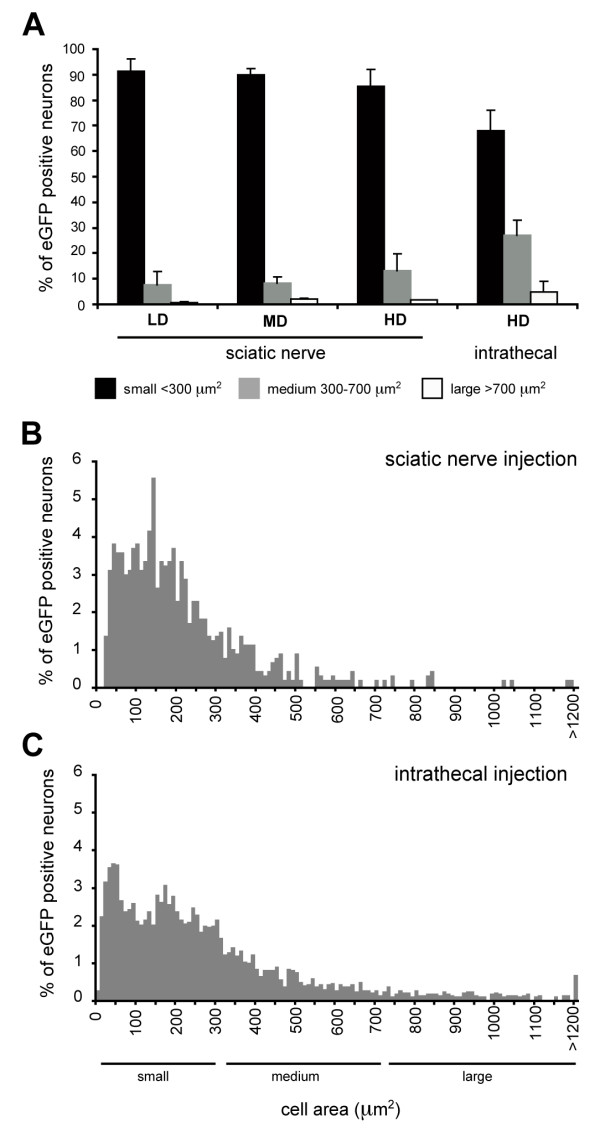
**Cell-size profile of eGFP-positive neurons in the L4 DRG following sciatic nerve and intrathecal injection**. (A) eGFP-positive neurons classified into small-sized (< 300 μm^2^, black bars), medium-sized (300-700 μm^2^, grey bars) and large-sized (>700 μm^2^, white bars) for the three doses of sciatic nerve injection and the intrathecal injection. Cell-size frequency histogram for eGFP-positive cell population three weeks following sciatic nerve (B) (n = 3, 858 eGFP-positive neurons measured) and intrathecal injection (C) (n = 5, 3146 eGFP-positive neurons measured) of 2.6 × 10^5 ^tu rAAV2/6.

We next characterized the transduction profile of the most efficient administration routes, the sciatic nerve and intrathecal deliveries. As primary sensory neurons in mice represent a diverse cell population that can be classified according to size [20]; small C-fiber polymodal nociceptive neurons < 300 μm^2^, medium-sized Aδ-fiber thermal and mechanical nociceptive neurons between 300 and 700 μm^2^, and large Aβ-fiber mechanoreceptive neurons and Aα-fiber proprioceptive neurons > 700 μm^2^, we have determined the distribution of cell size (μm^2^) for the eGFP-positive cells in relation to the total eGFP-positive cell population (Fig. 2A, 2B). Greater than 90% of eGFP-positive cell sizes corresponded to small to medium-sized cell types (<700 μm^2^) for sciatic nerve and intrathecal injection (Fig. 2C) suggesting transduction of nociceptive neurons.

**Figure 3:**
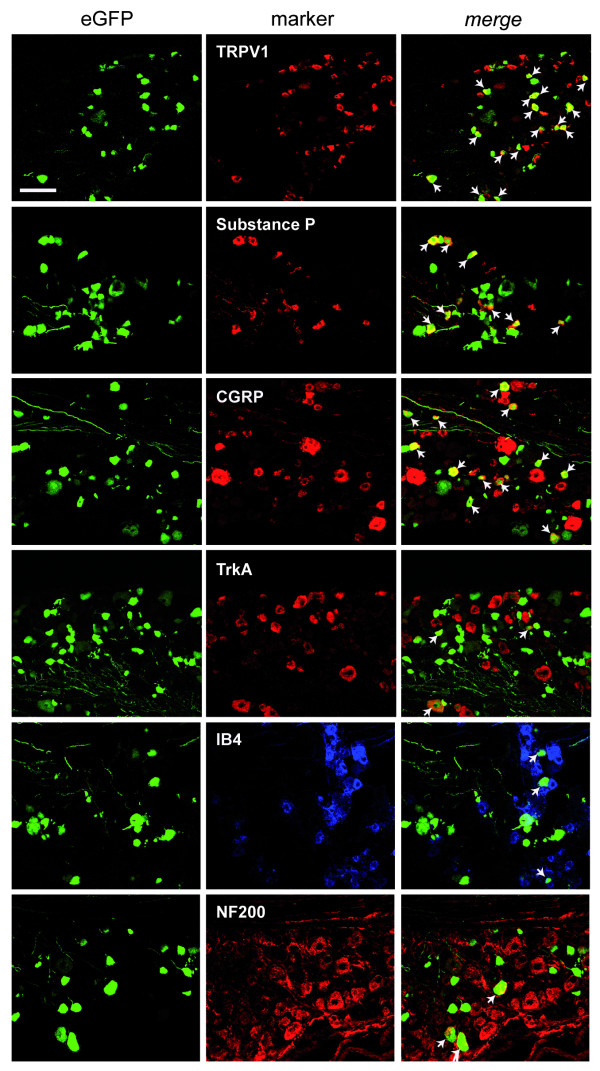
**Neuronal transduction pattern following sciatic nerve injection**. Representative confocal images demonstrating colocalization of eGFP-positive neurons with markers against neuronal subpopulations following injection of 2.6 × 10^5 ^tu rAAV2/6 into the sciatic nerve. TRPV1, Substance P, CGRP and TrkA for small peptidergic neurons, IB4 for non-peptidergic neurons and NF200 for large neurons. Scale bar: 100 μm.

**Figure 4:**
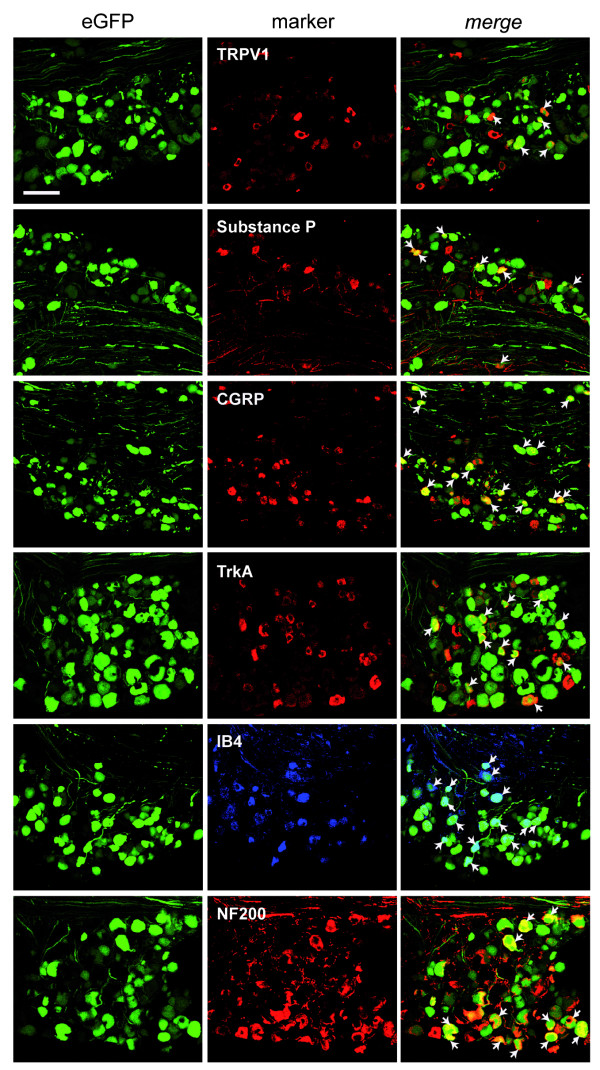
**Neuronal transduction pattern following intrathecal injection**. Representative confocal images demonstrating colocalization of eGFP-positive neurons in L4 DRG with markers against primary sensory neurons subpopulations following injection of 2.6 × 10^5 ^tu rAAV2/6 into the subarachnoid space. Scale bar: 100 μm.

Immunohistochemistry was next performed to further characterize the vector tropism for putative nociceptive neurons. L4 DRG sections were labeled with antibodies against markers of various neuronal populations and assessed for colocalization with eGFP (Fig. 3, 4). More eGFP-positive cells colocalized with transient receptor potential vanilloid 1 (TRPV1) than with neurofilament 200 (NF200) for both modes of delivery. Indeed NF200 colocalization with eGFP was rare, although, interestingly, was more frequent for the intrathecal than the sciatic nerve delivery. There was also colabeling with markers of the small peptidergic neurons, Substance P, α-calcitonin gene-related peptide (CGRP) and neurotrophic tyrosine kinase receptor type 1 (TrkA) and the non-peptidergic neuronal population, isolectin B4 (IB4) for both modes of delivery.

**Figure 5:**
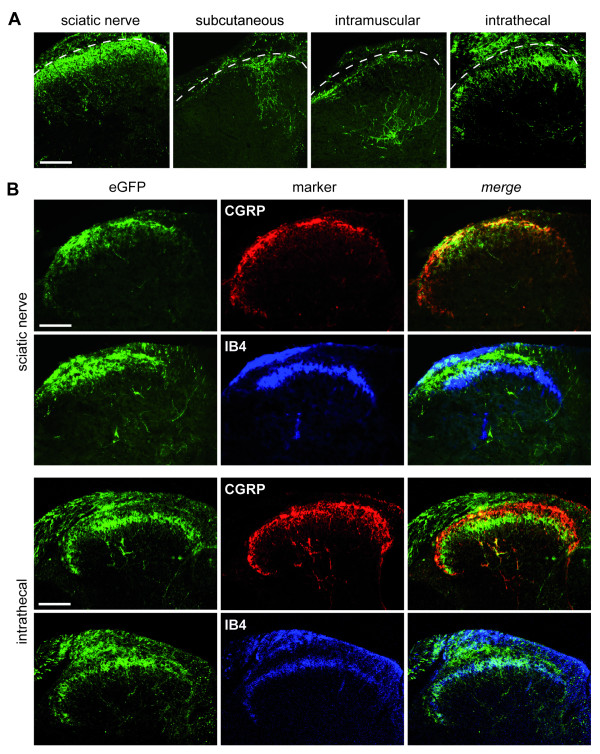
**Transduction of sensory fibers within the dorsal horn following rAAV2/6 delivery via different routes**. (A) eGFP expression in dorsal horn following sciatic nerve, subcutaneous, intramuscular, and intrathecal injection of 2.6 × 10^5 ^tu rAAV2/6. Scale bar: 100 μm. Confocal microscope images for CGRP (red, lamina I and II outer) and IB4 (blue, lamina II inner) against eGFP expression (green) in the dorsal horn following sciatic nerve (B) and intrathecal (C) injection. Scale bar: 50 μm.

The pattern of eGFP visualization within the spinal cord recapitulated this transduction of putative nociceptive neurons. eGFP-immunoreactive fibers were located almost exclusively within the superficial lamina I and II of the dorsal horn following sciatic nerve and intrathecal injections whereas subcutaneous and intramuscular administration transduced central terminals of DRG neurons within deeper laminae (Fig. 5A, 5B). Curiously, the transduced fibers following sciatic nerve delivery colocalized extensively with CGRP1 suggesting an enriched transduction of peptidergic neurons following this delivery route.

As glial cells have recently emerged as key contributors to chronic pain mechanisms [21], we examined whether sciatic nerve and intrathecal deliveries resulted in transduction of astrocytes and microglia in the spinal cord dorsal horn. No colocalization was observed with glial cell markers glial fibrillary acidic protein (GFAP; astrocytes) nor ionized calcium binding adaptor molecule 1 (Iba1; microglia). Similarly, the transduction of satellite cells within the DRG was not observed (see additional file 1: satellite and glial cells.tif). Large eGFP-positive cells (> 25 μm) were observed within the ventral horn and possibly corresponded to transduction of motor neurons via retrograde transport from the nerves (see additional file 2: eGFP anterior horn.tif).

### Intrathecal administration of rAAV2/6 results in transduction at remote sites within the nervous system and periphery of mice

**Figure 6:**
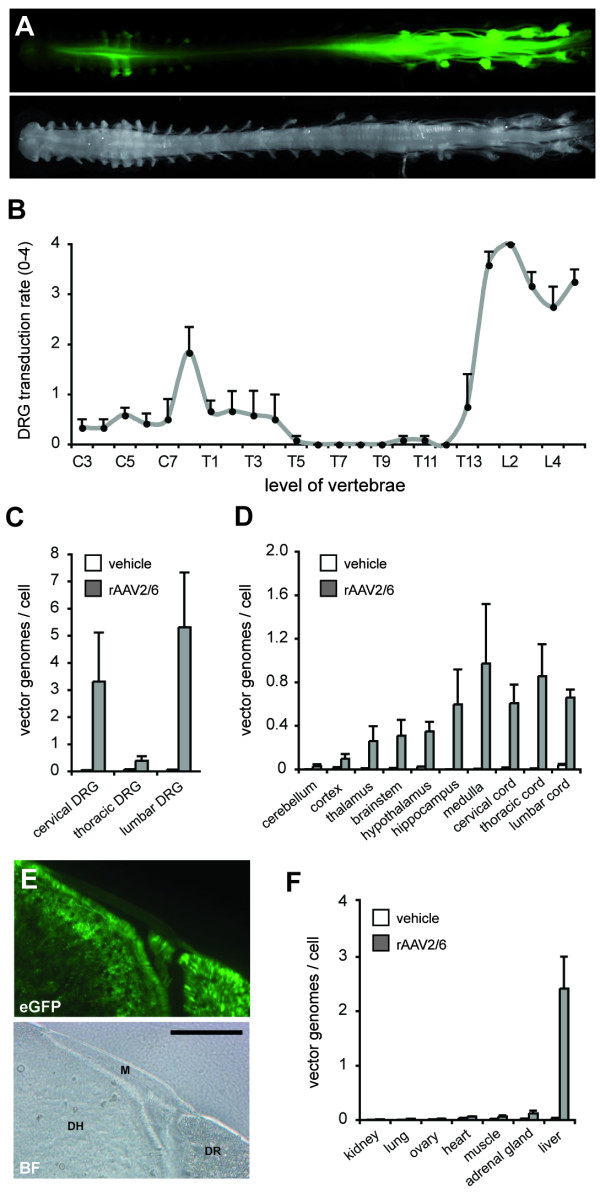
**Global transduction profile following intrathecal delivery of rAAV2/6**. C57Bl/6 mice were intrathecally injected with 2.6 × 10^5 ^tu rAAV2/6 and tissues analyzed for the vector dissemination. (A) Macroscopic epifluorescence of the whole spinal cord and intact DRG demonstrate transduction across lumbar, thoracic and cervical vertebral levels. (B) DRG were scored for eGFP expression (0 = zero, 1 = faint, 2 = weak, 3 = moderate and 4 = intense eGFP expression) (n = 6). qPCR analysis of vector genome copy number per cell in (C) DRG and (D) brain and spinal cord. (E) Native eGFP expression in spinal cord section containing intact meninges. eGFP is present in the dorsal horn and dorsal root but not within the subarachnoid space or membranes of the pia mater and arachnoid. M, meninges, DH, dorsal horn, DR, dorsal root. Scale bar: 50 μm. (F) qPCR against the rAAV2/6 genome in peripheral tissues.

Considering the unexpected observation that intrathecal delivery of rAAV2/6 resulted in relatively high levels of vector genomes in the liver (Fig. 1D), we next characterized the global spread of the vector following this mode of administration. Macroscopic analysis of eGFP epifluorescence from whole dissected spinal cord and DRG revealed significant transduction across the breadth of the rostral-caudal axis (Fig. 6A, 6B). Lumbar DRG were the most strongly transduced with peak transduction consistently occurring at L1 and L2 (n = 6). Surprisingly, cervical DRG expressed higher levels of eGFP-positive cells than the thoracic DRG which contained few eGFP-positive cells. qPCR against the vector genomes confirmed this transduction profile, with cervical, thoracic and lumbar DRG containing 3.3 ± 1.8, 0.4 ± 0.2 and 5.3 ± 2.0 vg/cell, respectively (Fig. 6C). The spinal cord and most of the regions of brain also contained the presence of vector genomes, although considerably less than the DRG (< 1 vg/cell) (Fig. 6D). Interestingly, the leptomeningeal cells that are bathed in the cerebrospinal fluid did not express eGFP (Fig. 6E). All peripheral tissues examined (except for the liver) contained negligible levels of vector genome (< 0.2 vg/cell) (Fig. 6F).

### DRG transduction efficiency is not altered in the SNI mouse model of neuropathic pain

In order to determine whether rAAV2/6-mediated gene transfer was applicable in mouse models of neuropathic pain, C57Bl/6 mice were intrathecally injected with the vector three days following nerve ligation in the spared nerve injury (SNI) model (n = 3 per group). No significant difference was observed between groups with sham and SNI-treated animals presenting 37 ± 8 and 48 ± 3 percent of eGFP-positive cells per total cell number in L4 and L5 DRG, respectively (*P *= 0.52) (see additional file 3: SNI transduction.tif). This finding demonstrates that rAAV2/6 can be used as a gene transfer tool in mechanistic studies for chronic pain.

### Mouse genetic background does not alter tropism of vector for nociceptive neurons

**Figure 7:**
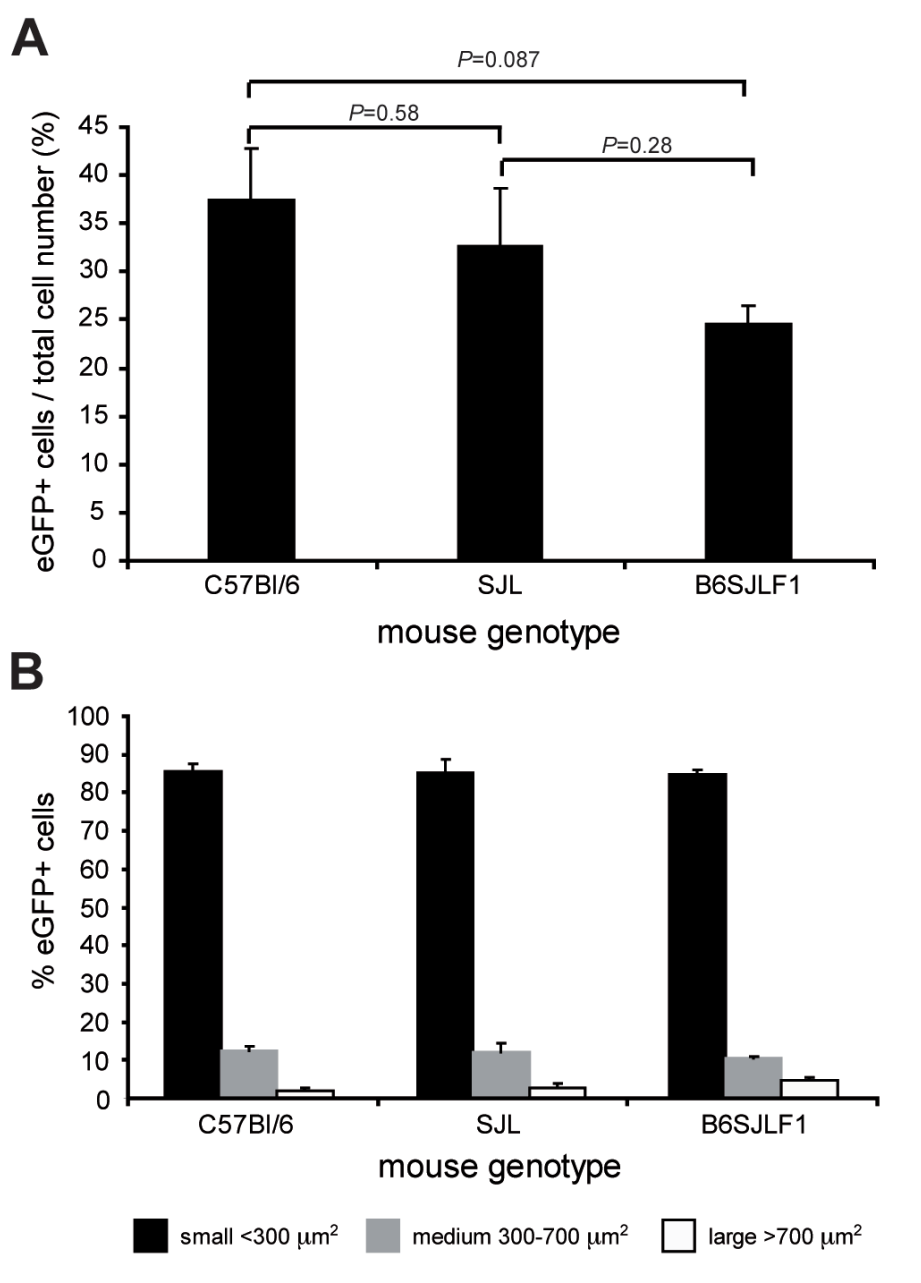
**Genetic background does not affect rAAV2/6 neuronal tropism**. DRG transduction was assessed in C57Bl/6, SJL and B6SJLF1 mice following sciatic nerve injection of 2.6 × 10^5 ^tu rAAV2/6. (A) Transduction rates within L4 DRG from the three different mice strains expressed as a percentage of eGFP-positive cells per total cell number (n = 3 per group). (B) Relative amounts of eGFP-positive neurons classified by somata size (small, medium, large).

The vector was administered to different strains of mice to determine whether the rAAV2/6 nociceptive neuron tropism was dependant on the genetic background of the host. C57Bl/6, SJL and B6SJLF1 mice were injected into the sciatic nerve with the high dose of rAAV2/6 and sacrificed three weeks later. eGFP expression was observed in between 25 and 35% of the total cell population in the DRG in accordance with previous results (Fig. 7A). There was a trend for an increased transduction of the C57BL/6 compared to the B6SJLF1 background although this was below significance (*P *= 0.087). The cell-size distribution of eGFP-positive cells was similar between strains supporting a consistent transduction profile (Fig. 7B). These results suggest that the rAAV2/6 tropism for nociceptive neurons is independent of mouse strain.

### Intravenous delivery of rAAV2/6 results in transduction of non-nociceptive DRG neurons

**Figure 8:**
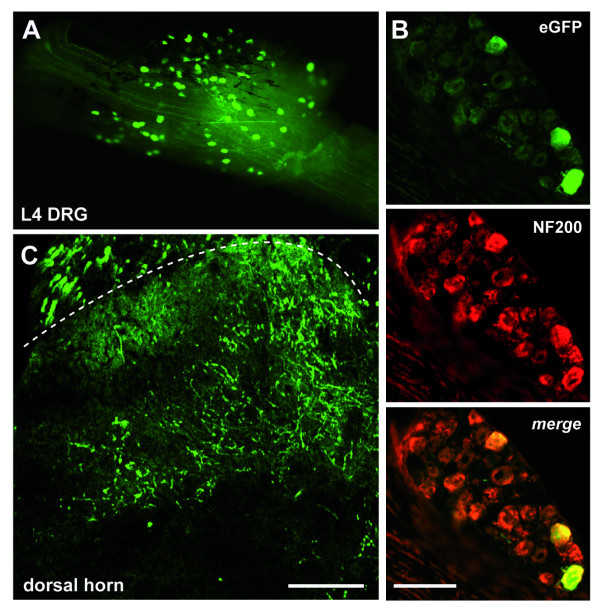
**eGFP expression in DRG and spinal cord following intravenous delivery of rAAV2/6**. (A) eGFP expression in whole L4 DRG following intravenous injection of 1.2 × 10^7 ^tu rAAV2/6. Colocalization against NF200 (B) and eGFP expression in the deep lamina of the dorsal horn (C) demonstrate predominantly non-nociceptive transduction. Scale bar: 100 μm.

Two reports have recently described transduction of fibers within the dorsal spinal cord following intravenous administration of rAAV [18,22]. To determine whether this mode of delivery results in transduction of cells within the DRG, we injected 1.2 × 10^7 ^tu rAAV2/6 into the tail vein of C57BL/6 mice. Three weeks following intravenous delivery we observed relatively low levels of eGFP expression in the DRG at all levels of the spine (L4 DRG shown in Fig. 8A). These cells colocalized predominantly with NF200 (Fig. 8B) and not with peripherin (data not shown). Similarly, only transduced fibers within the deeper lamina of the spinal cord were observed (Fig. 8C).

## Discussion

Although a considerable number of reports have described the use of HSV and adenovirus as gene transfer agents in the peripheral nervous system, both are derived from disease-causing viruses that express structural proteins capable of eliciting an immune response, invariably leading to short-term gene expression (< 2 months) and cytotoxicity [6,23,24]. rAAV is a non-inflammatory vector that provides efficient and long-term expression in neurons *in vivo *and whose parent wild-type virus has never been associated with disease. In the present study, we have examined the ability of rAAV serotype 6 to deliver a transgene to the dorsal root ganglia of mice following five different routes of administration. We have carefully estimated the percentage of cells transduced and characterized the transduction profile in terms of cell size and colocalization with neuronal markers. When combined or compared with transgenic approaches, this vector may provide valuable information in the mechanistic study of pain.

The ability of rAAV2/6 to transduce sensory neurons from the periphery was examined first as this vector has been reported capable of retrograde axonal transport [18,25]. Delivery into the triceps surae muscle of the hindlimb resulted in transduction of cells within the L4 DRG albeit at low levels. The transduction may have arisen through infection of intrafusal muscle fibers and Golgi tendon organs that are located in the muscle and send proprioceptive information to the spinal cord. This is in accordance with the observation that eGFP-positive fibers were located in the deeper lamina of the dorsal horn. Subcutaneous delivery of rAAV2/6 also resulted in low levels of eGFP-positive neurons in the L4 DRG. Curiously, transduction of the DRG following subcutaneous delivery has not been observed previously for rAAV, with rAAV serotype 2 (rAAV serotype 2 genome packaged in the serotype 2 capsid, rAAV2/2) failing to transduce DRG cells following subcutaneous injection into the hindpaw of rats [13]. This is not surprising as rAAV2/2 is inefficient at retrograde transport in comparison to rAAV2/6 [25].

Cutaneous and subcutaneous delivery of HSV has frequently been used to transduce sensory neurons of rats and mice as proof-of-principle for gene therapy of chronic pain [16,26]. We cannot compare the transduction observed here with previous reports as the vast majority has not quantified transduction efficiency. This is in part due to the use of secreted transgenic peptides that have had the capacity to exert paracrine functions on non-transduced cells. At least in one report, however, topical delivery of HSV was shown to achieve approximately 50% of total DRG neurons [15]. Despite the relatively lower transduction observed in the present study, subcutaneous delivery of rAAV2/6, with further optimization, may be an alternative to HSV for the delivery of secreted transgenic peptides towards human gene therapy of pain. Indeed, rAAV is the only viral vector currently being examined in clinical trials for neurological disease [27].

Transgene delivery to nociceptive neurons can also be used to explore the nature of changes that lead to chronic pain states. For example, HSV has been used to investigate signaling pathways that result in altered ion channel functions that have become hyper-responsive to channel activation and pain signaling [28]. Fink et al [29] have also used HSV to examine the role of TNFα signaling in chronic pain. With the goal of increasing the number of transduced nociceptive neurons that would be required for exploring the mechanisms of pain, we examined sciatic nerve and intrathecal routes administration of rAAV that have recently reported success in the transduction of rat DRG. Indeed, injection of rAAV2/6 into the sciatic nerve of C57BL/6 mice resulted in eGFP expression in more than 30% of the L4 DRG neurons. This is a substantial fraction of cells considering that the L4 DRG has afferent projections into the sciatic nerve and also other nerves of the lumbosacral plexus that were not injected (eg. pudendal nerve, obturator nerve, posterior cutaneous nerve of the thigh) that account for 40-50% of total L4/L5 DRG neurons [30]. Interestingly, the efficiency and profile of transduction was similar for the three mouse strains examined, suggesting that rAAV2/6 transduction of DRG neurons can be applied to the mouse regardless of the model being used.

The level of transduction observed in this study was similar to sciatic nerve delivery of rAAV2/2 in the rat [13,31,32]. The serotype 6 rAAV used in the present report has previously been delivered to the sciatic nerve of rats to result in retrograde transport to motor neurons, however, transduction in the DRG was not examined [25]. Other viral vectors have also been examined through this delivery route. HSV has resulted in efficient transduction of the L4 DRG, although declined dramatically after 3 weeks [8,33,34]. Adenovirus has also been administered to the sciatic nerve of rats and mice, although has led to transduction of predominantly Schwann cells on both accounts [35,36].

The cell-size profile of transduced cells was next characterized to determine whether nociceptors were efficiently targeted. Approximately 90% of eGFP-positive cells were small-sized (<300 μm^2^) corresponding to putative nociceptive neurons. As only one half the total DRG cell population of mice are less than 300 μm^2 ^[20], we can infer that there was a transduction preference for this small nociceptive cell population. Indeed, more frequent colocalization of eGFP was observed with markers of small DRG neurons (TRPV1, Substance P, CGRP, TrkA and IB4) than with large neurons (NF200) in the DRG. eGFP-positive fibers were present mainly in the superficial lamina of the spinal cord and colocalized extensively with CGRP1 (lamina I and II outer) and less with IB4 (lamina II inner) suggesting an enriched transduction of peptidergic neurons following this route of administration. Perhaps the apparent transduction preference of nociceptors is attributed to the degree of myelination that may impede entry of the virion into larger fibers following injection into the nerve. Further work would be required to confirm this.

Intrathecal administration into mice was next examined as this has previously been reported to result in efficient rAAV-mediated gene transfer to the DRG of rats [9,10]. To negate the need of performing a laminectomy in the relatively smaller rodent, a four centimeter catheter was inserted into the cistern magna and moved to the level of the lumbar spinal cord [37,38]. rAAV2/6 delivery resulted in the highest transduction observed in the present study, reaching approximately 60% of L4 DRG neurons. eGFP-positive cells colocalized with markers of nociceptive and non-nociceptive cells, however, upon analysis of cell area, there was a tendency for less small-sized neurons (<300 μm^2^) (*P *= 0.15) and more medium-sized neurons (300 - 700 μm^2^) (*P *= 0.18) than for the sciatic nerve delivery. Importantly, we found that transduction efficiency was unaltered following nerve ligation, demonstrating that rAAV2/6 can be used in models of neuropathic pain.

The results observed here in mice are similar to that obtained with rAAV serotype 8 (rAAV serotype 2 genome packaged in the serotype 8 capsid) in rats, that led to high levels of transduction of TRPV1, Substance P, CGRP and IB4 positive cells within the DRG [10]. Curiously, rAAV2/2 failed to transduce DRG cells following this mode of delivery [13] demonstrating different cellular tropisms among rAAV serotypes. Adenovirus has also been delivered intrathecally for chronic pain studies to result in efficient (although transient) expression of proteins within the cerebrospinal fluid, such as beta-endorphin [39] and interleukin-10 [40], although transduction was not examined in the DRG.

Although intrathecal delivery results in transduction of more DRG neurons, the sciatic nerve injection still has advantages for studies of neuropathic pain. Unlike intrathecal administration, injection of the sciatic nerve can be performed unilaterally, which allows the non/vehicle-treated side to serve as a useful internal control for behavior or histology. This specificity of the nerve injection is further exemplified by the finding that only this injection delivery route failed to result in spread of the vector into the blood stream, whereas intrathecal administration gave considerable transduction in the liver. Direct nerve injection can also be used to target injured versus non-injured nerves, for example, in the spared nerve injury model [41], where the spread of rAAV2/6 following intrathecal delivery would preclude this. Intrathecal administration may therefore be more suited to behavioral studies where the greater number of cells transduced may allow better discrimination of changes to pain perception in the whole animal.

Intravenous administration via the tail vein was also examined as this had previously resulted in transgene expression in the fibers of the spinal cord dorsal horn with rAAV2/8 [22] and rAAV2/6 [18]. This mode of delivery resulted in eGFP-positive cells within the DRG at lumbar, thoracic and cervical levels of spinal cord, and colocalized with the marker of non-nociceptive neurons, NF200. This novel mode of transduction may have resulted through infection of nerve endings within the muscle. This follows the reasoning that 1) skeletal muscle is highly vascularized and 2) the transduction pattern in the deeper lamina of the spinal cord is similar to that observed after intramuscular rAAV2/6 delivery.

One caveat of this study has been that eGFP expression was examined only at the designated three week time point. Previously we have reported expression in neurons at least 8 weeks following injection with the rAAV2/6 vector [19] and also observed stable expression for at least 5 months in spinal cord motor neurons (unpublished observations). Taken together with the multitude of studies demonstrating long-term transgene expression for rAAV in neurons, we believe that the rAAV2/6-mediated transgene expression in nociceptive neurons would also be stable through time.

## Conclusion

We have examined rAAV2/6-mediated *in vivo *gene transfer to neurons of mouse DRG through five routes of delivery. Not only have we matched transduction efficiencies achieved with other vectors, but we have mapped the transduction profiles in terms of cell-size, cell marker expression and fiber localization within the spinal cord. We propose that rAAV2/6 is an efficient gene transfer tool in rodents that can be used for the mechanistic study and targeted molecular evaluation of proteins in the area of neuropathic pain. Furthermore, the diversity of transduction depending upon mode of delivery can be exploited to differentially target mechanisms in primary sensory neuron populations.

## Materials and methods

### Virus production and titration

rAAV2/6 was produced by cotransfection of the pAAV-CMV shuttle plasmid coding for eGFP with the pDF6 packaging plasmid into the 293AAV cell line [42]. Cell lysates were subjected to purification by high pressure liquid chromatography on the HiTrap Heparin column (GE Healthcare Bio-Sciences AB, Uppsala, Sweden) 48 hr later. The obtained viral suspension was concentrated with Centricon Plus-20 (Regenerated Cellulose 100,000 MWCO (Millipore, Billerica, MA)) and the suspension medium replaced with PBS. The infectivity (transduction units per volume) of the rAAV2/6 was determined by flow cytometry for direct eGFP fluorescence. The percentage of eGFP-positive cells was quantified 48 hr after infection of 293T cells with respect to uninfected control cells. The number of transduction events was calculated using the Poisson equation.

### Animals and vector administration

All manipulations were performed in six-week-old male C57BL/6 mice (Charles River Lab, L'Abresle, France) except for the strain comparison experiment that also used SJL and B6SJLF1 mice (Charles River Lab). All procedures were approved by the Committee on Animal Experimentation for the canton of Vaud, Switzerland, in accordance with Swiss Federal Law on Animal Welfare and the guidelines of the International Association for the Study of Pain [43]. Animals were housed under a 12 h light/dark cycle and had free access to food and water. Vector injections were performed under anesthesia with either ketamine-xylazine (120 mg/kg ketamine, 10 mg/kg xylazine) or 1.5% isoflurane (Abott, Baar, Switzerland), except intravenous injections which were performed without anesthesia.

rAAV2/6 was injected through five different routes. For intramuscular injections, an incision was made on the lower leg to expose the triceps surae muscle and 30 μL of the vector were injected using a 30-gauge (30 G) Hamilton syringe (Bonaduz, Switzerland). For subcutaneous injections, 10 μL of vector solution were injected in the glabrous plantar skin territory of the left hindpaw using a 30 G Hamilton syringe. For sciatic nerve injections, an incision was made on the thigh to expose the quadriceps femoral muscles which were then carefully separated to expose the nerve. The vector was manually delivered in 6 μL using a 30 G Hamilton syringe after which the muscle and skin were closed in two distinct layers with 5.0 silk threads and wound clips, respectively. Three doses of virus were used for the intramuscular, subcutaneous and sciatic nerve delivery: 2.6 × 10^4^, 8.0 × 10^4 ^and 2.6 × 10^5 ^tu of rAAV2/6.

Intrathecal administration was performed in two parts and used 2.6 × 10^5 ^tu of rAAV2/6. Mice were first anesthetized with ketamine-xylazine and then placed vertically with their head fixed in a stereotaxic frame. An incision was made in the base of the neck to expose the groove in the nuchal crest. An incision was made (1 - 2 mm) in the cisternal membrane to a depth such that cerebrospinal fluid leaked out. A 4 cm 32 G intrathecal catheter (Micor CS-1, Micor Inc, Allison Park, PA, USA) was then slowly inserted in the direction of the lumbar spinal cord and skin was closed by suture around the catheter. The mice were then allowed to recover. Any animals that presented motor or gait disturbance 2 hr following the surgery were sacrificed. In the second part, mice that did not present motor abnormalities were anesthetized with isoflurane and the vector (6 μL) was administered. The catheter was flushed with 6 μL of PBS and was then removed and mice allowed to recover. For intravenous injections, 200 μL containing 1.2 × 10^7 ^tu of the vector in PBS were injected into the tail vein using a 25-gauge Terumo^® ^syringe (Leuven, Belgium). Control animals received PBS or no injection.

### Spared nerve injury

Adult C57BL/6 mice were operated for SNI using 1.5% isoflurane as described previously [44]. Briefly, after exposure of the sciatic nerve, the common peroneal and tibial nerves were ligated with 5.0 silk suture and transected while the sural nerve was left intact. Muscle and skin were closed in two distinct layers with 5.0 silk thread and wound clips. Sham surgery was performed the same although without nerve ligation. Mice were injected intrathecally three days post-surgery with 2.6 × 10^5 ^tu of rAAV2/6-eGFP and sacrificed for analysis three weeks later.

### Immunohistochemistry

Three weeks after injection, animals were terminally anesthetized with sodium pentobarbital and transcardially perfused with saline, followed by 4% paraformaldehyde in PBS. DRG and spinal cord segments were dissected and post-fixed at 4°C for 90 min or 3 hr, respectively. Tissues were then transferred to 20% sucrose in PBS overnight and mounted on dry ice the following day in cryoembedding fluid (Tissue-Tek; Sakura Finetek, Zoeterwoude, Holland). Dissected DRG and spinal cords were cryosectioned at 12 and 20 μm thickness, respectively, and mounted directly onto slides. The eGFP signal on spinal cord sections was increased using a rabbit anti-eGFP antibody (1:500, Molecular Probes, CA). Direct eGFP or anti-eGFP signals were colabeled using the following antibodies: rabbit anti-α-calcitonin gene-related peptide (CGRP, 1:5000, Peninsula Laboratories, San Carlos, CA), rabbit anti-vanilloid receptor subtype 1 (TRPV1; 1:200; gift from GlaxoSmithKline, Middlesex, United Kingdom), rat anti-Substance P (1:400, BD Bioscience, Basel, Switzerland), mouse anti-neurofilament 200 (NF200, 1:500, Sigma, Basel, Switzerland), rabbit anti-ionized calcium binding adaptor molecule 1 (Iba1, 1:200, Wako Pure Chemical Industries, Osaka, Japan) and rabbit anti-glial fibrillary acidic protein (GFAP, 1:1000, DakoCytomation, Glostrup, Denmark). Primary antibodies were revealed by the appropriate Cy3-conjugated secondary antibodies (1:300, Jackson ImmunoResearch, Suffolk, UK). Non-peptidergic neurons were labeled using biotinylated griffonia simplicifolia Isolectin B4 (IB4) (1:200 in DRG, 1:50 in spinal cord, Vector Laboratories, Burlingame, CA) coupled to AMCA-conjugated Streptavidin (1:200; Jackson ImmunoResearch). Except for the monoclonal NF200 antibody that utilized the mouse-on-mouse (MOM) kit (Vector Laboratories, Burlingame, CA), standard fluorescent immunohistochemistry techniques were used for all protocols. Briefly, sections were blocked for 30 min at room temperature in PBS 10% serum and 0.3% triton X-100 and then incubated overnight at 4°C with primary antibody or biotinylated IB4 diluted in PBS 5% serum and 0.1% Triton X-100. Sections were washed in PBS and incubated for 90 min at room temperature with corresponding Cy3-conjugated secondary antibody, or AMCA-conjugated streptavidin, diluted in PBS serum 1% and 0.1% Triton X-100. Slides were then washed again and mounted with Mowiol mounting medium (Calbiochem, Gibbstown, NJ).

### qPCR

Total DNA and RNA was isolated from tissues using QIAGEN DNA/RNA Mini Kit (QIAGEN, Valencia, CA). The amount of vector genomes per cell for each tissue was determined by qPCR with the iQ SYBR Green Supermix (Bio-Rad, Hercules, CA) using standard conditions. Primers were targeted against the beta-globin intron of the rAAV2/6 vector (forward, TCTTATCTTCCTCCCACAGC; reverse, GTCACTCTTGGCACGGGGAA) and the mouse chemokine (C-C) motif receptor 5 (CCR5) of the host genome (forward, GTCCTCCTCCTGACCACCTTC; reverse, GCAGCAGTGTGTCATTCCAAG). Vector genomes copies for each sample were quantified according to the plasmid standard curve and were expressed for 2N genomes according to the CCR5 copy number measured in the same sample as described previously [18]. The relative levels of eGFP mRNA were quantified by reverse transcription qPCR. Isolated RNA was used as a template to generate cDNA via StrataScript^® ^First-Strand Synthesis System (Stratagene, La Jolla, CA). Relative amounts of eGFP transcripts between different tissues was determined by qPCR with primers targeting eGFP (forward, TGACCCTGAAGTTCATCTGCACCA; reverse, TCTTGTAGTTGCCGTCGTCCTTGA), normalized using primers against glyceraldehyde-3-phosphate dehydrogenase (GAPDH) (forward, TCCATGACAACTTTGGCATTG; reverse, CAGTCTTCTGGGTGGCAGTGA). Amplifications were performed using the 7900HT Fast Real Time PCR System (Applied Biosystems Inc, Foster City, CA). Dissociation curves confirmed amplification of solely one PCR product for all qPCR reactions.

### Counting and statistics

Direct eGFP fluorescence detection in whole DRG was performed using an epifluorescent microscope (AxioPlan, Zeiss, Feldbach, Switzerland). Scoring of DRG following intrathecal injection was performed using the following qualitative criteria (0 = zero, 1 = faint, 2 = weak, 3 = moderate and 4 = intense eGFP expression). Colocalization was performed using a Zeiss laser scanning confocal microscope (LSM 510 Meta, Zeiss). L4 DRG from three to four animals were analyzed per condition except for the SNI evaluation that used both L4 and L5 DRG for the quantification. To evaluate transduction rates, a slide from the first 5 was randomly selected and then every fifth DRG section was selected from the series of consecutive serially cut sections (4 - 5 sections per DRG). In each selected section, the number of direct eGFP fluorescent cells was counted and transduction efficiency was expressed as the percentage of the total cell number. To construct profile size distribution histograms, we measured the area of eGFP-positive cells using the AxioVision LE Rel.4.3 Software (Zeiss). Neurons were classified in three groups: small (<300 μm^2^), medium (300-700 μm^2^) and large (>700 μm^2^) neurons as described previously [20]. Data are represented as mean ± SEM. Differences between groups were compared using Student's *t *test. Statistical analyses were performed using JMP statistical software (version 5.01, SAS institute, Cary, NC). A *P *value ≤ 0.05 was considered statistically significant.

## Abbreviations

rAAV: recombinant adeno-associated virus; CGRP: α-calcitonin gene-related peptide; DRG: dorsal root ganglia; eGFP: enhanced green fluorescent protein; GFAP: glial fibrillary acidic protein; HSV: herpes simplex virus; IB4: isolectin B4; Iba1: ionized calcium binding adaptor molecule 1; IT: intrathecal; NF200: neurofilament 200; qPCR: quantitative PCR; rAAV2/2: rAAV serotype 2 genome packaged in the serotype 2 capsid; rAAV2/6: rAAV serotype 2 genome packaged in the serotype 6 capsid; rAAV2/8: rAAV serotype 2 genome packaged in the serotype 8 capsid; SNI: spared nerve injury; TrkA: neurotrophic tyrosine kinase receptor type 1; TRPV1: transient receptor potential vanilloid 1.

## Competing interests

The authors declare that they have no competing interests.

## Authors' contributions

CT initiated the study and drafted the manuscript. He prepared and titrated the virus for the experiments and performed the intramuscular and intravenous injections. He assisted in the other surgeries, performed the qPCR and contributed to the analysis and interpretation of the data. MP performed the spared nerve injury, the subcutaneous and sciatic nerve injections and assisted in other virus injections. She harvested tissues from the animals, performed the immunohistology, cell quantification and scoring of whole DRG. She assisted in the preparation of the figures and contributed to the analysis and interpretation of the data. ATB developed and performed the technique of intrathecal administration of the virus and assisted in the perfusion and dissection of animals. PA provided the facilities and materials for the production of the virus, advice on the experimental protocols, and edited the manuscript. ID oversaw all stages of the study and edited the manuscript. She contributed to the analysis and interpretation of the data and is primarily responsible for the integrity of the data and the main conclusions.

## Supplementary Material

Additional file 1 **eGFP does not colocalize with neurons or glia in the spinal cord dorsal horn nor with satellite cells in the DRG**. eGFP expression does not colocalize with antibodies against neurons (NeuN), microglia (Iba1) and astrocytes (GFAP) in the spinal cord dorsal horn following intrathecal delivery of rAAV2/6. eGFP expression in the dorsal horn is confined to NF200 or peripherin labeled neurons and not to smaller satellite cells as depicted with DAPI. Scale bar: 50 μm. Click here for file

Additional file 2 **eGFP expression in the spinal cord anterior horn following intrathecal and sciatic nerve delivery of 2.6 × 10^5 ^tu rAAV2/6**. eGFP-positive cells have large cell bodies (> 25 μm) and colocalize with CGRP. CGRP is expressed by a subset of motor neuron pools and is a non-specific marker for this cell type in the spinal cord. Scale bar: 100 μm. Click here for file

Additional file 3 **DRG transduction efficiency is not altered in the SNI mouse model of neuropathic pain**. C57Bl/6 mice were intrathecally injected with rAAV2/6 three days following nerve ligation in the spared nerve injury (SNI) mouse model of neuropathic pain. No significant difference in eGFP transduction rate was observed between sham and SNI-treated animals (n = 3 per group) (*P *= 0.52). Click here for file
