# Supplementary figures and images for: Recombinant adeno-associated virus serotype 6 (rAAV2/6)-mediated gene transfer to nociceptive neurons through different routes of delivery
Source: Mol Pain. 2009 Sep 8;5:52. doi: 10.1186/1744-8069-5-52 (PMC2747840; doi:10.1186/1744-8069-5-52)

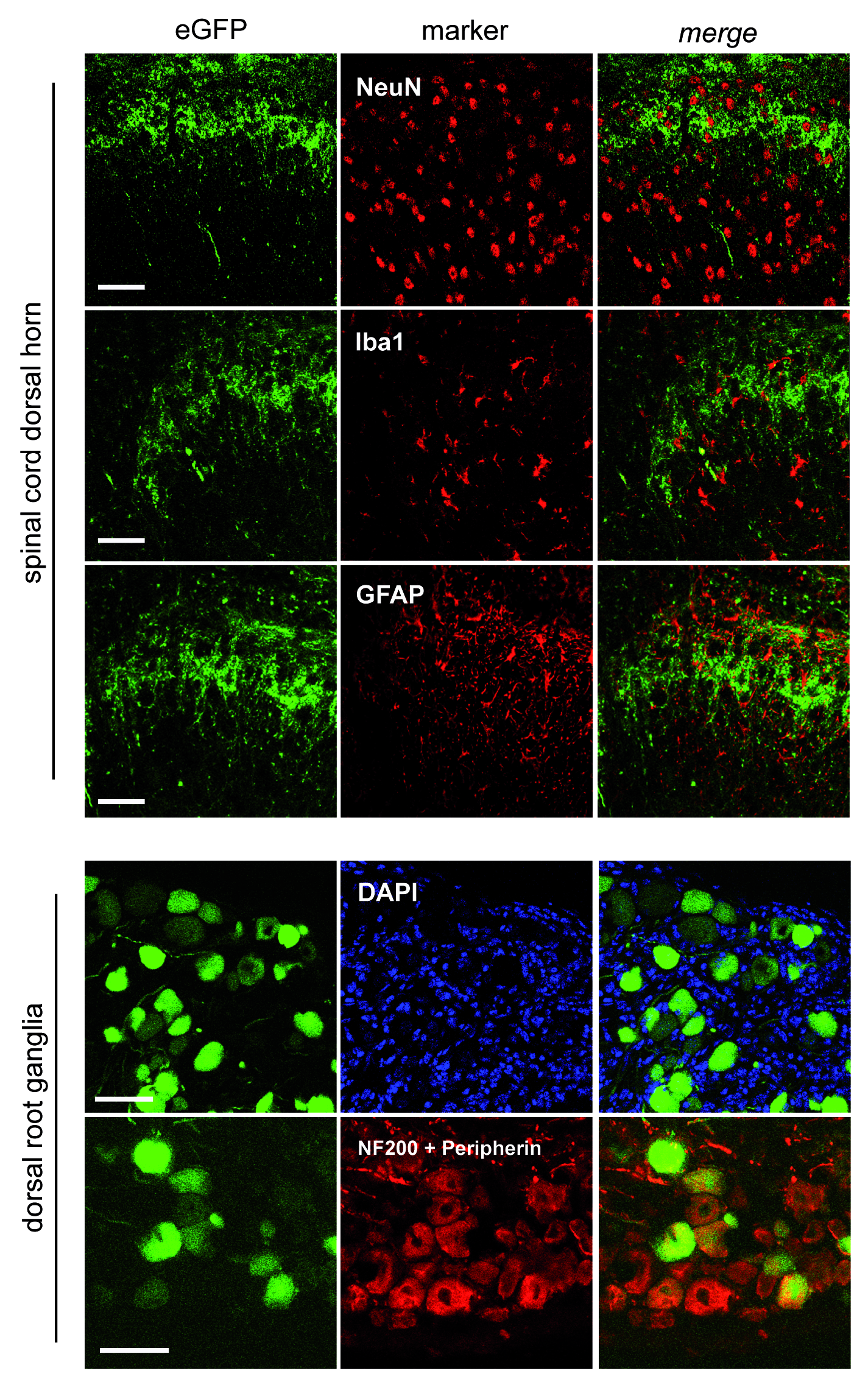

Supplement: Additional file 1 — eGFP does not colocalize with neurons or glia in the spinal cord dorsal horn nor with satellite cells in the DRG. eGFP expression does not colocalize with antibodies against neurons (NeuN), microglia (Iba1) and astrocytes (GFAP) in the spinal cord dorsal horn following intrathecal delivery of rAAV2/6. eGFP expression in the dorsal horn is confined to NF200 or peripherin labeled neurons and not to smaller satellite cells as depicted with DAPI. Scale bar: 50 μm. [file 1744-8069-5-52-S1.tiff]

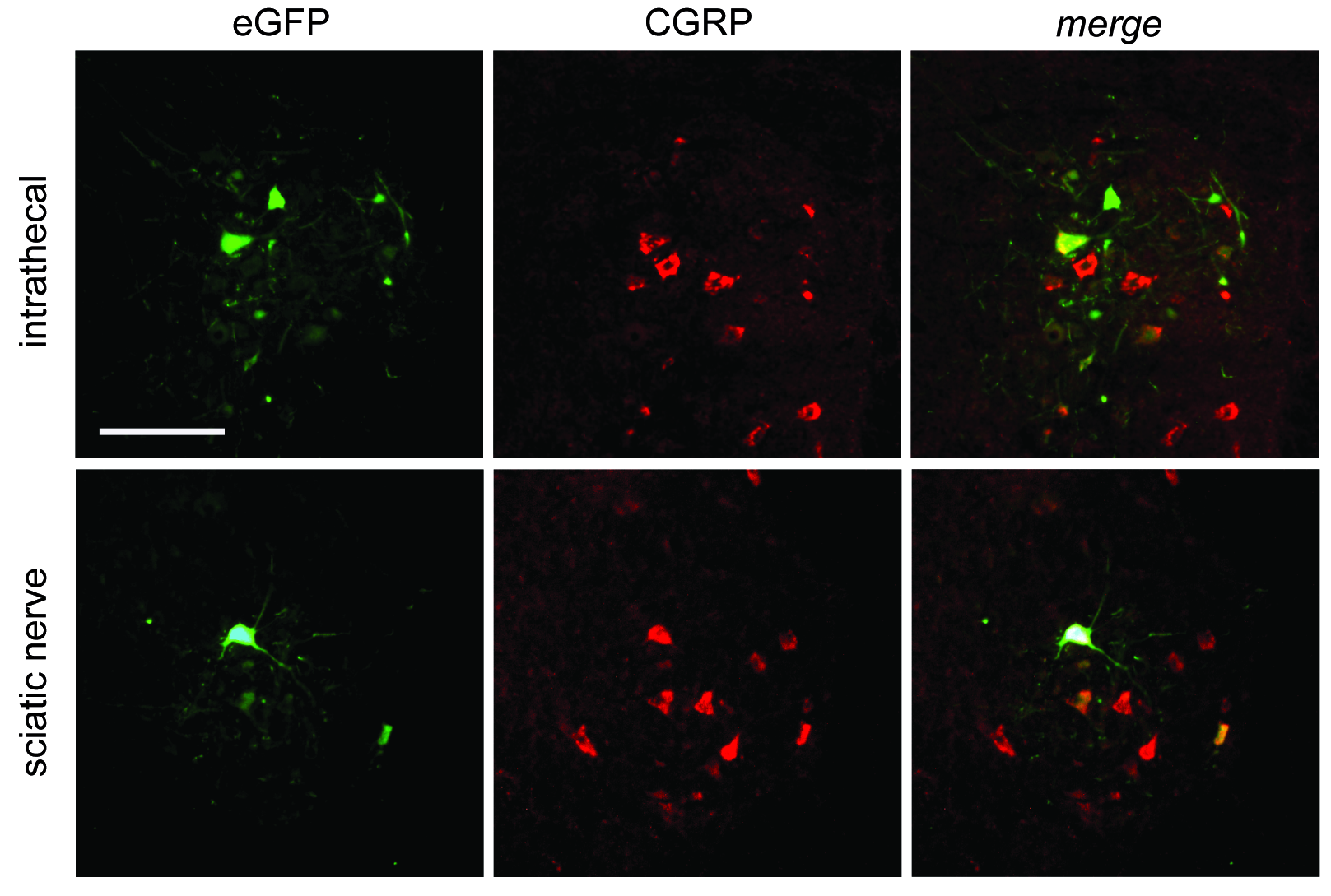

Supplement: Additional file 2 — eGFP expression in the spinal cord anterior horn following intrathecal and sciatic nerve delivery of 2.6 × 10^5^ tu rAAV2/6. eGFP-positive cells have large cell bodies (> 25 μm) and colocalize with CGRP. CGRP is expressed by a subset of motor neuron pools and is a non-specific marker for this cell type in the spinal cord. Scale bar: 100 μm. [file 1744-8069-5-52-S2.tiff]

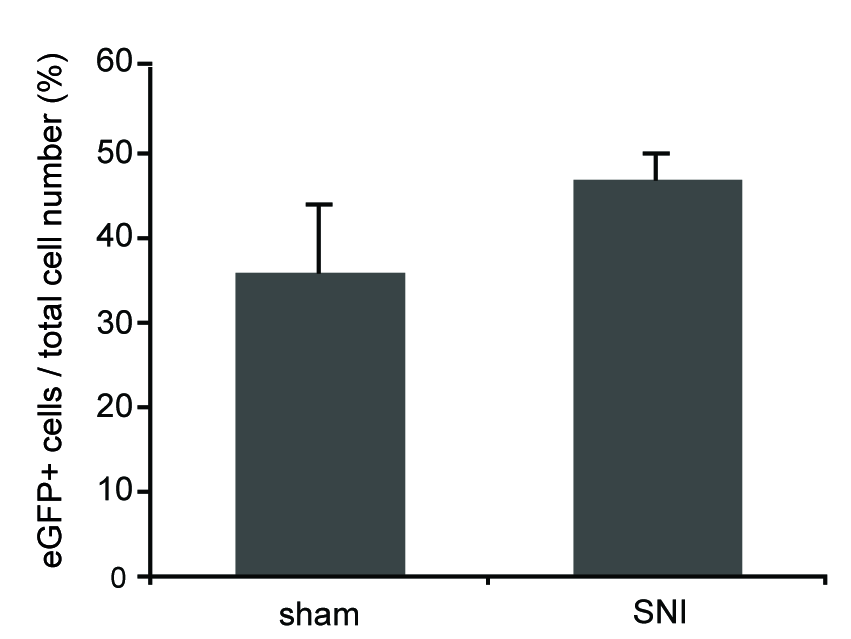

Supplement: Additional file 3 — DRG transduction efficiency is not altered in the SNI mouse model of neuropathic pain. C57Bl/6 mice were intrathecally injected with rAAV2/6 three days following nerve ligation in the spared nerve injury (SNI) mouse model of neuropathic pain. No significant difference in eGFP transduction rate was observed between sham and SNI-treated animals (n = 3 per group) (P = 0.52). [file 1744-8069-5-52-S3.tiff]
